# Quantitative considerations about the size dependence of cellular entry and excretion of colloidal nanoparticles for different cell types

**DOI:** 10.1007/s40828-021-00159-6

**Published:** 2022-01-25

**Authors:** Yanan Kang, Leroy Marwin Nack, Yang Liu, Bing Qi, Yalan Huang, Ziyao Liu, Indranath Chakraborty, Florian Schulz, Abdullah A. A. Ahmed, Mirco Clavo Poveda, Fereshta Hafizi, Sathi Roy, Marina Mutas, Malte Holzapfel, Carlos Sanchez-Cano, K. David Wegner, Neus Feliu, Wolfgang J. Parak

**Affiliations:** 1 Center for Hybrid Nanostructures (CHyN) and Fachbereich Physik and Chemie, Universität Hamburg, Hamburg, Germany; 2 Present Address: Key Laboratory of Biological Nanotechnology of National Health Commission, Xiangya Hospital, Central South University, Changsha, Hunan China; 3 Fraunhofer Center for Applied Nanotechnology (CAN), Hamburg, Germany; 4 Mildred Scheel Cancer Career Centre Hamburg, Universitätklinikum Hamburg-Eppendorf, Hamburg, Germany; 5 Center for Cooperative Research in Biomaterials (CIC biomaGUNE), Basque Research and Technology Alliance (BRTA), San Sebastián, Spain; 6 Division Biophotonics, Federal Institute of Materials Research and Testing (BAM), Berlin, Germany

**Keywords:** Cell proliferation, Exocytosis, Gold nanoparticles, Luminescent nanoparticles, Quantum dots, Fluorescence, Uptake studies, Nanoparticle degradation

## Abstract

Most studies about the interaction of nanoparticles (NPs) with cells have focused on how the physicochemical properties of NPs will influence their uptake by cells. However, much less is known about their potential excretion from cells. However, to control and manipulate the number of NPs in a cell, both cellular uptake and excretion must be studied quantitatively. Monitoring the intracellular and extracellular amount of NPs over time (after residual noninternalized NPs have been removed) enables one to disentangle the influences of cell proliferation and exocytosis, the major pathways for the reduction of NPs per cell. Proliferation depends on the type of cells, while exocytosis depends in addition on properties of the NPs, such as their size. Examples are given herein on the role of these two different processes for different cells and NPs.

## Introduction

Nanoscience has led to advances in physics, materials science, and biology, thereby impacting on applications in electronics, photovoltaic devices, biosensing, and bioimaging [1, 2]. Therefore, increasing amounts of different nanomaterials can be found in our daily life, not only in electronics such as smartphones, tablets, and television screens, but also in wall paints, clothing, and cosmetics. This constant exposure of our body to nanomaterials, whether unwanted or intended, has fueled research on the interaction of nanoparticles (NPs) and cells, providing new insights into their cytotoxic effects and helping to improve the design of nanomaterials to minimize hazardous effects on human health [3–6]. In addition, development of nanomedicines has also occurred for therapeutic or diagnostic purposes. Their high surface-to-volume ratio, numerous functionalization possibilities, and particular optoelectronic and magnetic properties make nanomaterials attractive for use in applications such as theranostics and bioimaging [1]. However, to achieve efficient use in those applications, it is necessary to be able to control and manipulate the number of NPs within the cells during a certain time window. Therefore, knowledge about cell uptake, metabolism, and excretion of NPs must be obtained [7].

There are many studies about the uptake of colloidal NPs by cells, in particular in vitro, but also in vivo. These have mostly focused on the influence of parameters such as the size, shape, surface charge, and surface functionality of the NPs on their intracellular accumulation kinetics [8, 9]. However, many fewer studies have been carried out on what happens to the NPs once they are inside cells [10]. NPs may be, for example, intracellularly degraded [11], which in turn may affect their cytotoxic potential. Usually, the degradation products of NPs will result in increased cytotoxicity as compared with intact NPs, e.g., by releasing toxic ions [12–14]. There are also some reports about the excretion of NPs by exocytosis, in which, for example, a relationship with size was observed for transferrin-coated gold NPs with faster exocytosis rates for smaller NPs. However, their shape may also play a role, as rod-shaped NPs have been reported to exhibit faster clearance from cells than spherical-shaped NPs [7]. Similar to cell uptake, the type of functional groups on the surface of the NPs can influence their exocytosis [15–17]. Despite those examples, the total body of work on exocytosis is limited, and many fewer publications exist on the fate of internalized NPs [16, 18–21].

Before discussing several studies that included quantitative analysis of the fate of internalized NPs, we want to point out that a key point when studying the interaction of NPs with cells is the quantitative aspect [22]. It is of utmost importance to know how the amount of NPs per cell varies with time. This is of particular interest in the above-mentioned scenarios of an environmental spill or exposure of humans to nanomedicines. Long-term toxic effects will depend on the rate at which NPs can be cleared by cells. For this reason, fundamental quantitative studies on their fate are important. We thus explain, based on a few recent studies, how quantitative studies of the cell uptake and clearance of NPs can be conducted using mass spectrometry and fluorescence-based techniques. Based on these studies, we discuss the dependence of exocytosis on the size of the NPs, and the role that cell proliferation plays in the reduction of NPs per cell.

## Proliferation versus exocytosis

In general, two different major pathways exist to reduce the amount of NPs (or their degradation products) per cell after their internalization by cells. To simplify the discussion, the NPs are first considered as one entity, neglecting their degradation/decomposition. The first pathway is cell proliferation, where cell division results in the internalized NPs being passed to both daughter cells, thus reducing the number of NPs per cell [23]. The second pathway for NP reduction in cells is exocytosis, where NPs are excreted by the cells via exocytotic vesicles [16]. Depending on the type of cells and also the properties of the NPs, the respective importance of these two mechanisms varies [24].

**Fig. 1:**
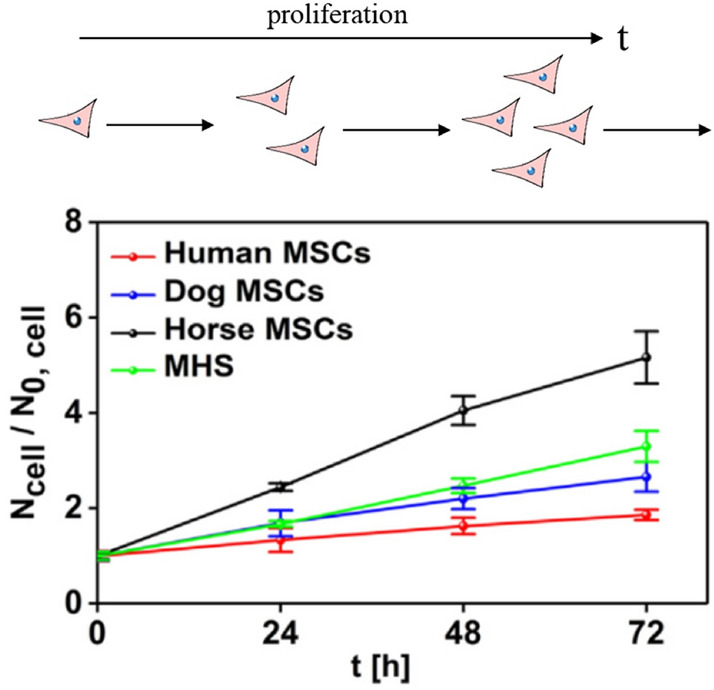
Comparison of cell division of primary mesenchymal stem cells (MSCs) of different origins and murine alveolar macrophages (MHS) over time, where *N*_cell_ is the number of cells in culture versus the initial amount of seeded cells (*N*_0,cell_). The time interval after which *N*_cell_/*N*_0,cell_ doubles is the average time a cell needs for one proliferation cycle. Image taken with permission from Sun et al. [24]

First, we discuss cell proliferation. As mentioned above, upon cell division, internalized NPs are passed to both daughter cells [23], thus reducing the number of NPs per cell. One can imagine that the proliferation rate may impose a limit on the amount of NPs that can be internalized per cell. As shown in Fig. 1, the time a cell needs to divide is typically on the time scale of days, whereas endocytosis occurs on the time scale of hours [24]. The longer the time needed for a cell to proliferate in comparison with the time needed to endocytose NPs, the more NPs can accumulate per cell. In contrast, in the (unrealistic) hypothetical case that the time for one proliferation cycle would be much faster than the time needed to endocytose NPs, cells would divide faster than they could uptake NPs, thus the amount of NPs per cell would remain minimum. In general, the cell proliferation rate is highly cell dependent [24]. Splitting of NPs between cells is thus cell type dependent, so for quantitative fate studies of NPs, the proliferation time of the investigated cells should be determined.

**Fig. 2:**
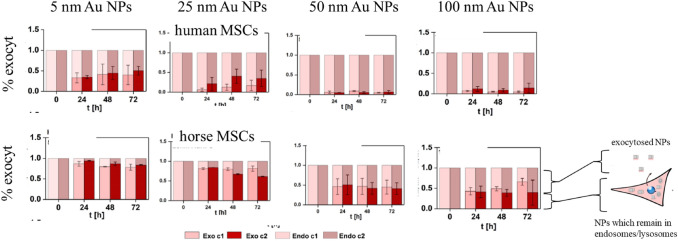
Percentage of exocytosed differently sized gold NPs in human primary mesenchymal stem cells (MSCs; **A**) and horse MSCs (**B**) at different time points. Image taken with permission from Sun et al. [24]. The NPs used here had Au cores with diameters from 5 to 100 nm and were all coated with the same polymer, viz. poly(isobutylene-*alt*-maleic anhydride)-*graft*-dodecyl (PMA)

The second pathway for NP reduction in cells is exocytosis, where NPs are excreted through exocytotic vesicles by cells and thus the number of intracellular NPs decreases. To perform a quantitative estimation of such NP reduction via exocytosis, residual NPs after the exposure first need to be removed from the extracellular medium (i.e., there are no longer NPs in the extracellular medium). Then, the rise in the NPs in the extracellular medium due to exocytosis must be measured at different time points. This can be done, for example, using elemental analysis techniques such as inductively coupled plasma mass spectrometry (ICP-MS). This enables a quantification of the amount of NPs excreted from the cells by exocytosis. Figure 2 shows exemplarily the exocytosis efficiency of differently sized gold NPs for human and horse stem cells [24]. It can be clearly seen that, independent of the cell type, the 5-nm gold NPs had higher exocytosis rates than the 100-nm gold NPs, which is supported by earlier studies [7, 25]. This study thus suggests that smaller NPs (here 5 nm diameter) are exocytosed to a much greater extent than larger NPs (here 100 nm).

The results shown in Figs. 1 and 2 indicate that, after endocytosis, the amount of NPs in the cells is determined by two factors: first, how fast the cells proliferate, and second, how large the NPs are and thus the role of exocytosis.

## The advantage of mass-balanced studies

**Fig. 3:**
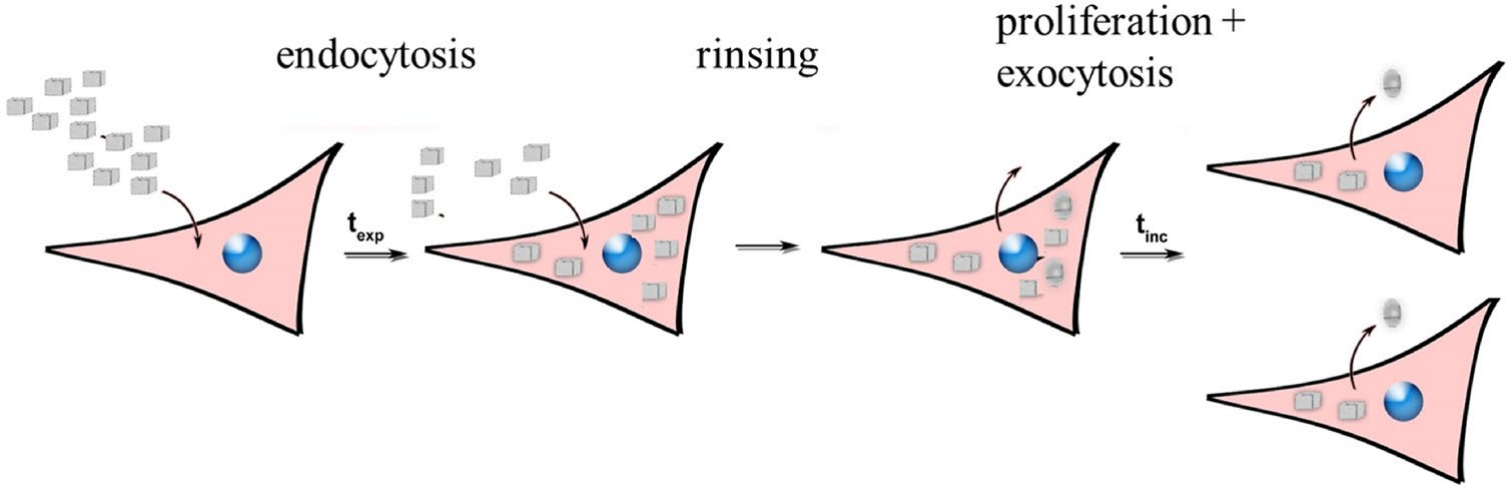
Scheme of fate studies. Cells are exposed to NPs for the exposure time *t*_exp_, after which excess NPs are removed from the cell medium by rinsing with fresh medium. Then, after a further incubation time *t*_inc_, the proliferation rate and the intra- and extracellular NP concentration are determined. Image taken with permission from Liu et al. [26]

Based on the finding that both proliferation as well as exocytosis are important to determine the amount of NPs per cell, the assay shown in Fig. 3 was developed for quantitative investigation of the fate of internalized NPs. Monitoring the amounts of exocytosed NPs is only one part of the story; It is also necessary to quantify the remaining intracellular NPs, which will provide greater insight into the fine balance between proliferation and exocytosis and allow study of the kinetics. The necessary experimental steps are shown in Fig. 3.

**Fig. 4:**
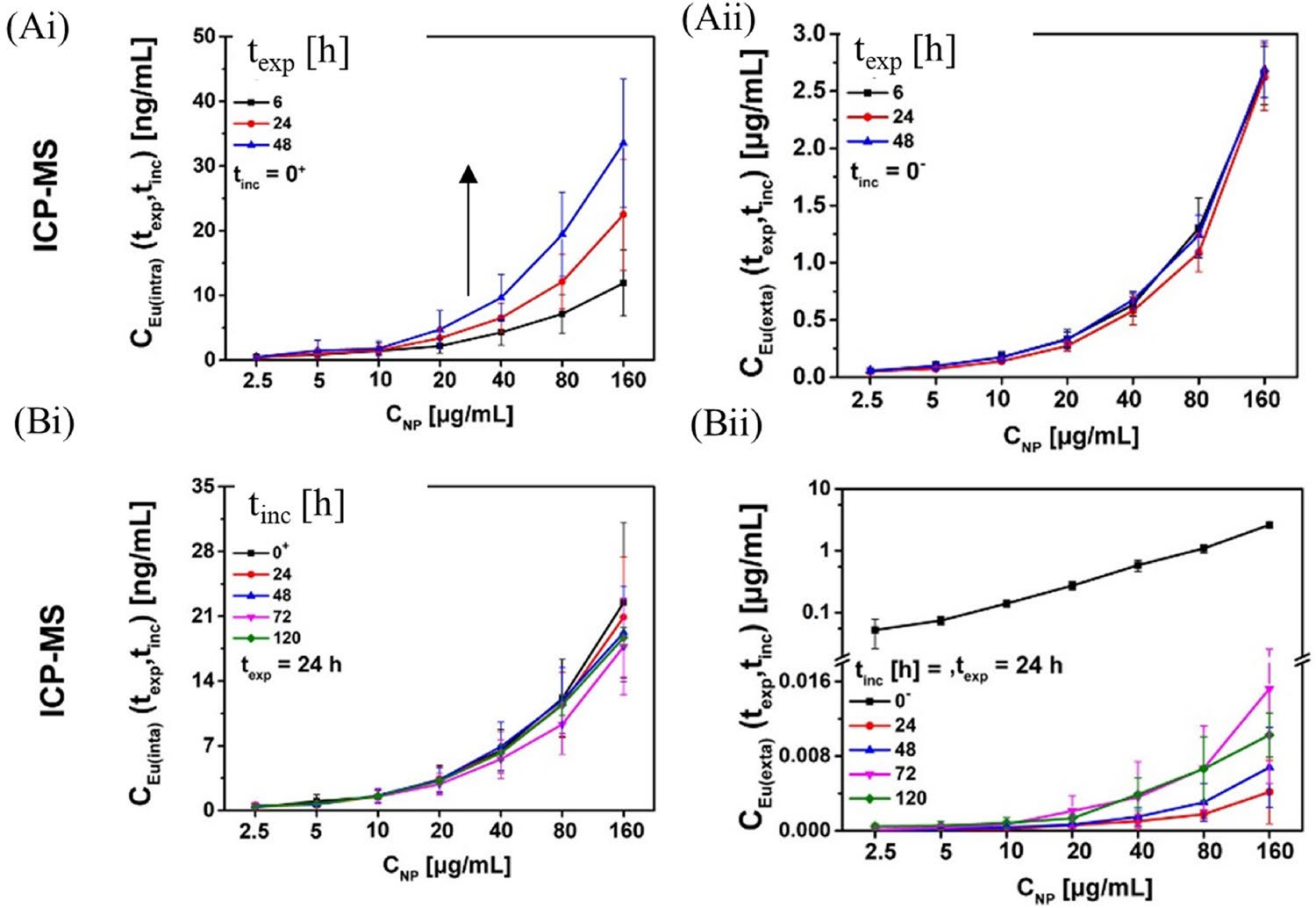
**A** Uptake of NPs over time in presence of NPs in the extracellular medium, and **B** loss of NPs over time when free NPs had been removed from the extracellular medium, both as functions of the NP concentration *C*_NP_. The absolute amounts of (i) intracellular NPs and (ii) NPs in the extracellular medium are reported. Image take with permission from Liu et al. [26]. These data were obtained with HeLa cells and Eu- and Bi-doped GdVO_4_ NPs of around 35 nm core diameter, coated with the polymer polyacrylic acid (PAA)

This scheme was used to investigate the fate of luminescent Eu- and Bi-doped GdVO_4_ NPs [27] of around 35 nm core diameter in adherent cell cultures [26]. The NP concentrations determined in the cells and the extracellular medium over time are shown in Fig. 4. In a first step, the uptake of NPs from the extracellular medium was quantified using ICP-MS. The expected time-dependent increase of intracellular NPs was observed (Fig. 4Ai), whereas the extracellular NP concentration remained almost unchanged as shown in Fig. 4Aii. This is a good example of how, in most exposure conditions, there is an “infinite” reservoir of extracellular NPs, i.e., the uptake is not limited by the availability of extracellular NPs in the medium. In a second step, the free NPs were removed from the extracellular media, then the time-dependent concentration of NPs inside cells and in the extracellular medium was measured. The obtained quantitative data are shown in Fig. 4Bi, ii. Interestingly, the amount of intracellular NPs remained almost constant over time (Fig. 4Bi), indicating that, in this case, exocytosis was not the major pathway for NP loss from the cells. Nevertheless, a slight increase of the extracellular NP concentration in the cell medium was observed (Fig. 4Bii), which should be due to exocytosis. However, the amount of exocytosed NPs was very small compared with the number of NPs that remained internalized in the cells. This low extent of exocytosis can be explained by the size of the NPs of ca. 35 nm, which according to the data shown in Fig. 2 leads to less exocytosis than for smaller NPs of 5 nm diameter, for which massive exocytosis has been reported. However, if one does not analyze the total amount of internalized NPs, but instead the amount of NPs internalized per cell, this amount of internalized NPs per cell decreases over time. This is due to proliferation, i.e., the almost constant amount of remaining internalized NPs is distributed among an increasing number of cells, thus the amount of NPs per cell is reduced. It has been pointed out that it is very important to be aware of how different methods for determining the amount of internalized NPs operate. Flow cytometry, for example, is a single-cell-based method and thus quantifies the amount of NPs per cell. Mass spectrometry (e.g., ICP-MS) is an ensemble-based measurement detecting the total amount of NPs in the cells. Thus, to determine the amount of NPs internalized per cell from ICP-MS data, the total amount of internalized NPs must be normalized by the actual number of cells in solution, which increases over time because of proliferation [26]. In summary, the main loss of internalized NPs over time in the example described in Fig. 4 is due to proliferation, e.g., the redistribution of internalized NPs among an increasing number of cells. The absolute amount of internalized NPs remaining stays almost unchanged, while only a small fraction is exocytosed. The NPs thus remain intracellular and are not cleared from cells [26]. Such mass-balanced studies, i.e., measuring the intracellular as well as the extracellular concentration of NPs over time, offers a convenient internal control over experimental errors; for example, where the sum of the intracellular and extracellular amounts of NPs does not remain constant over time, NPs must have been lost during experimental procedures (e.g., washing steps), which would bias a quantitative analysis.

## Taking into account NP degradation

**Fig. 5:**
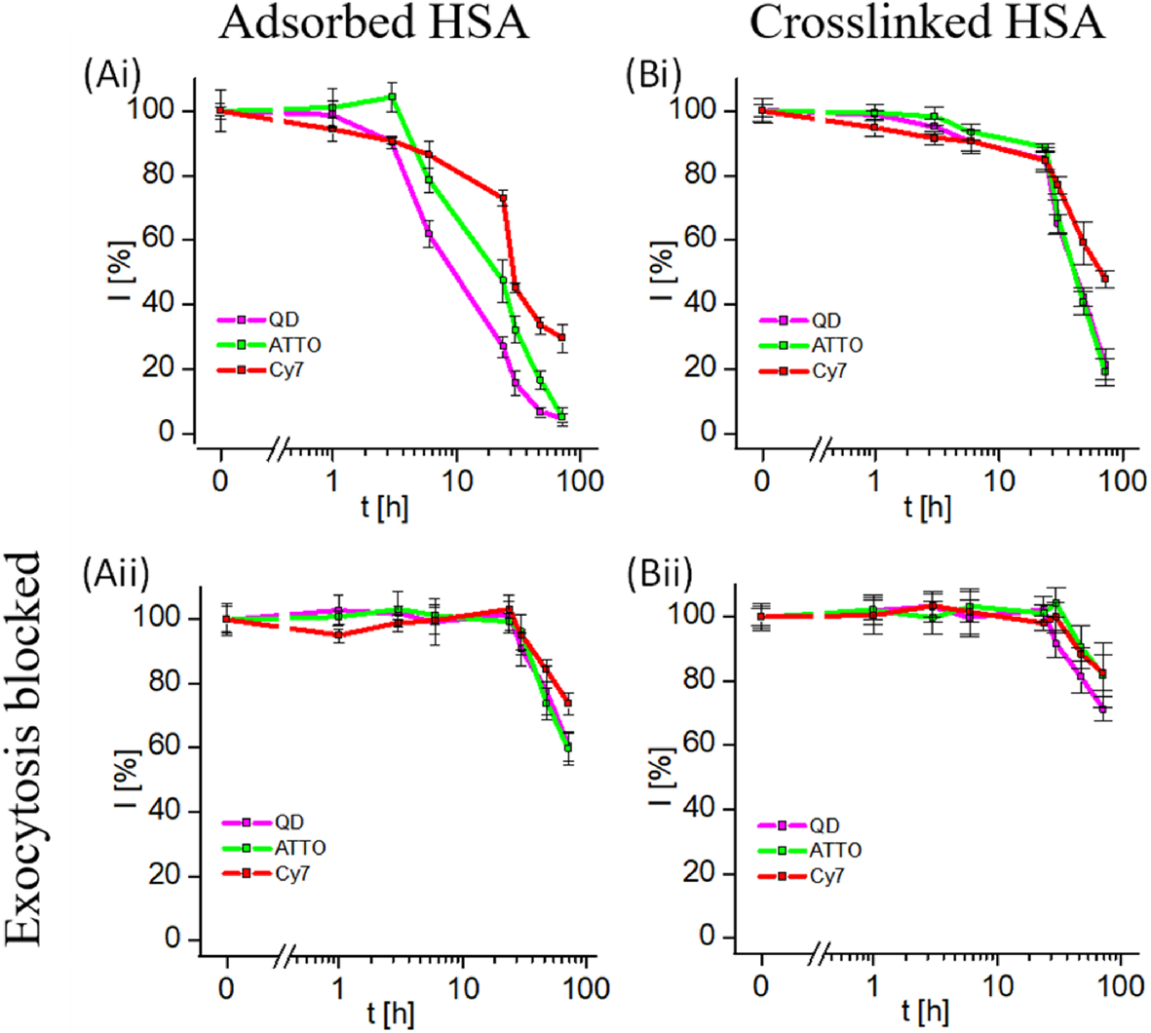
Three different compartments of quantum dots were fluorescence labeled by different colors: quantum dots (QDs) with their intrinsic fluorescence, the polymer surface coating with the organic fluorophore ATTO (ATTO488; ATTO-TEC GmbH, #AD488-91) [36], and human serum albumin as model protein with the organic fluorophore Cy7 (Sulfo-Cyanine7 NHS ester, Lumiprobe, #25320). The reduction of intracellular fluorescence after free NPs in the extracellular medium around cells with endocytosed NPs had been removed by rinsing. Image taken with permission from Carrillo-Carrion et al. [36]. These data were recorded with HeLa cells and CdSe/ZnS NPs with a core diameter of around 5.5 nm, coated with the polymer poly(isobutylene-*alt*-maleic anhydride)-*graft*-dodecyl (PMA), which was fluorescence labeled with ATTO

In the mass-balanced uptake and fate study illustrated in Fig. 4, NPs were regarded as a static entity; i.e., ions released from the NPs inside endosomes/lysosome would stay together (i.e., colocalize) with the NPs, either intracellularly or extracellularly. However, in a more realistic scenario, degradation products from the NPs may have a different fate than the NPs themselves. To monitor such processes, different parts of NPs can be marked with different labels. Fluorescence-based techniques are, for example, a versatile tool to study the degradation of fluorescent NPs in the cellular environment [28]. One of the most popular types of fluorescent NPs is semiconductor nanocrystals (quantum dots, QDs), which exhibit properties such as sharp and size-tunable emission bands. Their nanometric size and good photostability makes them good labels for bioimaging or biosensing [1, 29, 30]. A convenient method to render QDs biocompatible is to overcoat them with an amphiphilic polymer [31, 32]. Once in a biological environment, a protein corona is formed on their surface, which also impacts on their interaction with cells [33, 34]. Thus, the description of a “complete” NP must take into account at least three different entities: the NP core (here QDs), the surface coating, and the corona of adsorbed proteins [35]. The fate of such complex NP structures within the cellular environment can be studied by using different colors of fluorescence for the different compartments and fluorescence microscopy. For this purpose, fluorescent labels to encode the polymer shell and the protein corona made by human serum albumin (HSA) have been used [36]. Together with the intrinsic fluorescent QD core of around 5.5 nm diameter, these hybrid structures thus had three differently fluorescent components, which enabled them to investigate the fate of these three compartments independently in cell cultures. Similar to the studies illustrated in Fig. 4, after endocytosis of QDs, the remaining free QDs were removed from the medium by rinsing, and the intracellular fluorescence of the three different NP compartments was monitored over time by using flow cytometry. To distinguish between exocytosis- and proliferation-caused reduction of intracellular fluorescence, the experiments were carried out without (Fig. 5Ai) and with (Fig. 5Aii) the presence of an exocytosis blocker. As seen in Fig. 5Aii, when exocytosis was blocked, the three different NP compartments remained jointly intracellular, while only after > 10 h was there a loss in intracellular fluorescence. This loss in fluorescence, which was identical for all three different NP compartments, was caused by proliferation. From these experimental data, it cannot be concluded whether the NPs remained structurally intact, but even if parts of the polymer shell or adsorbed proteins detached from the NP cores, they would have remained together in the same endosomes/lysosomes and could not have been excreted by the cells via exocytosis (which was blocked). The time > 10 h after which intracellular fluorescence started to reduce could be reasonably explained by proliferation (cf. the data in Fig. 1). When cells divide, endosomes/lysosomes are split between the daughter cells, thus the kinetics of fluorescence loss would be the same for the three different NP compartments. In contrast, without the presence of the exocytosis blocker, there was a different kinetics of fluorescence loss for the three different NP compartments (Fig. 5Ai), which was also faster for all three compartments than the proliferation-related kinetics. This fast component of intracellular fluorescence loss can thus be ascribed to exocytosis. In contrast to the larger NPs illustrated in Fig. 4 (ca. 35 nm core diameter), for the small QDs (ca. 5.5 nm core diameter) exocytosis was an efficient pathway for the reduction of the number of intracellular NPs per cell over time. In addition, one can see that the NPs decomposed inside endosomes/lysosomes; i.e., parts of the surface coating and adsorbed proteins came off the NP cores and were thus exocytosed with a different kinetics. When the proteins were chemically cross-linked to the polymer coating, the NPs remained intact; i.e., all three NP components were exocytosed with the same kinetics (Fig. 5B).

## Discussion

**Fig. 6:**
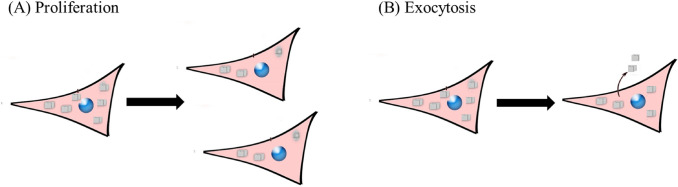
Sketch of **A** proliferation and **B** exocytosis, the two pathways by which the amount of NPs per cell can decrease

The presented examples describe how to perform quantitative measurements to investigate the cellular entry and excretion of NPs using ICP-MS and fluorescence-based techniques. Two main pathways must be considered to explain the reduction of NPs per cell over time after all NPs in the extracellular medium have been removed, viz. cell proliferation and exocytosis. Proliferation depends on the cell type used; no NPs are secreted to the extracellular medium, but the amount of NPs per cell is diluted due to cell division. Exocytosis of NPs, i.e., their secretion to the extracellular medium, shows strong size dependence, where smaller NPs are exocytosed more quickly than larger NPs, as shown in the sketch in Fig. 6.

One can thus consider two extreme cases. Firstly, for larger NPs and rapidly proliferating cells, the main loss of internalized NPs per cell over time will be due to cell proliferation, i.e., the redistribution of internalized NPs among an increasing number of cells, whereby the total amount of intracellular NPs remains almost constant without NP clearance from cells [26] but the amount of NPs per cell decreases due to the increasing number of cells. In contrast, for small NPs and slowly proliferating cells, exocytosis would be the main factor determining the decrease of the amount of NPs per cell over time. To separate these two processes, mass-balanced studies are required, i.e., recording the number of NPs inside cells as well as in the extracellular medium. After NP removal from the extracellular medium, NPs found after some time in the extracellular medium must be due to exocytosis. Monitoring of the NP concentration in the extracellular medium thus allows exocytosis to be monitored. Measuring the amount of intracellular NPs per cell, on the other hand, is a convenient approach to monitor proliferation. In case no extracellular NPs are detected, any decrease in the amount of intracellular NPs per cell over time is due to proliferation.

In principle, the arguments discussed herein are general, i.e., do not depend on the type of NP, in particular whether the NPs are degradable. If the NPs can degrade intracellularly (for this topic, we refer to the reviews [10, 35, 37]), upon proliferation the degradation products would still be passed to the daughter cells. However, as the degradation products will be smaller than the original NPs (while other physicochemical properties such as hydrophobicity may also change), the rate of exocytosis will change. While the experimental examples discussed herein are based on two-dimensional cultures of adherent cells, in principle the argument that both proliferation and exocytosis must be considered when analyzing the time-dependent intracellular NP dose holds also true in in vivo scenarios. However, naturally, proliferation as well exocytosis rates will be different in tissue.

Cell proliferation may actually vary significantly between different cell types [38, 39]. This might potentially influence treatment with NP-based pharmaceutical agents. Would administered NPs in a tumor be diluted faster by the highly prolific malignant cells than by less prolific cells, thus reducing their efficiency? This is an interesting question, which however is complicated to address in vivo. Also, exocytosis may vary significantly between different cell types. Would exocrine and endocrine secretory cell types use the secretory granules for exocytosis of NPs? This could be relevant for making extracellular membrane-coated NPs. Exosomes are one subclass of extracellular vesicles (EVs), which are lipid-based vesicles secreted by cells [40, 41], and which can be considered as natural NPs. By feeding cells with artificially made NPs (such as the Au NPs, Eu- and Bi-doped GdVO_4_ NPs, and CdSe/ZnS NPs illustrated in Figs. 2, 4, and 5), upon exocytosis these NPs will be coated with cellular membranes [42]. Better understanding of the kinetics of NP exocytosis may thus help the production of extracellular vesicle-coated NPs.
